# 9-PAHSA Improves Cardiovascular Complications by Promoting Autophagic Flux and Reducing Myocardial Hypertrophy in Db/Db Mice

**DOI:** 10.3389/fphar.2021.754387

**Published:** 2021-11-15

**Authors:** Yan-Mei Wang, Shou-Ling Mi, Hong Jin, Qi-Lin Guo, Zhong-Yu Yu, Jian-Tao Wang, Xiao-Ming Zhang, Qian Zhang, Na-Na Wang, Yan-Yan Huang, Hou-Guang Zhou, Jing-Chun Guo

**Affiliations:** ^1^ Department of Geriatrics of Huashan Hospital, National Clinical Research Center for Aging and Medicine, Department of Translational Neuroscience, Jing’an District Centre Hospital of Shanghai, State Key Laboratory of Medical Neurobiology and MOE Frontiers Center for Brain Science, Institutes of Brain Science, Fudan University, Shanghai, China; ^2^ Department of Cardiology, Zhongshan Hospital, Fudan University, Shanghai, China; ^3^ Shanghai Stomatological Hospital & Institutes of Biomedical Sciences, Fudan University, Shanghai, China

**Keywords:** diabetic cardiovascular complications, autophagy, 9-PAHSA, myocardial hypertrophy, vascular calcification

## Abstract

Atherosclerotic cardiovascular disease is a common and severe complication of diabetes. There is a large need to identify the effective and safety strategies on diabetic cardiovascular disease (DCVD). 9-PAHSA is a novel endogenous fatty acid, and has been reported to reduce blood glucose levels and attenuate inflammation. We aim to evaluate the effects of 9-PAHSA on DCVD and investigate the possible mechanisms underlying it. Firstly, serum 9-PAHSA levels in human were detected by HPLC-MS/MS analysis. Then 9-PAHSA was synthesized and purified. The synthesized 9-PAHSA was gavaged to db/db mice with 50 mg/kg for 4 weeks. The carotid arterial plaque and cardiac structure was assessed by ultrasound. Cardiac autophagy was tested by western blot analysis, electron microscope and iTRAQ. The results showed that 9-PAHSA, in patients with type 2 diabetes mellitus (T2DM), was significantly lower than that in non-diabetic subjects. Administration of 9-PAHSA for 2 weeks reduced blood glucose levels. Ultrasound observed that continue administration of 9-PAHSA for 4 weeks ameliorated carotid vascular calcification, and attenuated myocardial hypertrophy and dysfunction in db/db mice. Electron microscopy showed continue 9-PAHSA treatment significantly increased autolysosomes, while dramatically decreased greases in the myocardial cells of the db/db mice. Moreover, iTRAQ analysis exhibited that continue 9-PAHSA treatment upregulated BAG3 and HSPB8. Furthermore, western blot analysis confirmed that 9-PAHSA down-regulated Akt/mTOR and activated PI3KIII/BECN1 complex in diabetic myocardium. Thus, 9-PAHSA benefits DCVD in diabetic mice by ameliorating carotid vascular calcification, promoting autophagic flux and reducing myocardial hypertrophy.

## Introduction

Diabetic cardiovascular complication (DCVC) is a common and severe complication of diabetes mellitus. DCVC is characterized by diastolic dysfunctions, followed by systolic impairment and left ventricle abnormalities. It often leads to high mortality with properties of severer infarction and poorer prognosis than those without diabetes (Voulgari et al., 2010; Tarquini et al., 2011; Kovacic et al., 2014). The pathogenesis of DCVC is complex and multifactorial, and hyperglycemia and inflammation are two of the important factors. Besides, accumulating evidence has recently suggested that autophagy play a key role in the pathophysiology of metabolic dysregulation and related cardiovascular complications (Xu and Brink, 2016; Luo et al., 2019). Autophagy-lysosomal pathway is a major cellular clearance machinery, which maintains metabolic homeostasis by degradation and clearance of long-lived or damaged proteins. Moreover, autophagy in cardiomyocytes play a key role in mediating hyperglycemia-induced cell dysfunction and damage. To this end, autophagy offers promising targets for novel strategies to prevent and treat DCVC. Targeting autophagy using pharmacological or natural agents is an emerging strategy for DCVC.

Palmitic-acid-9-hydroxy-stearic-acid (9-PAHSA) is a recently discovered endogenous lipid that is highly elevated in the adipose tissue of transgenic mice overexpressing glucose transporter 4 (GLUT4) (Shepherd et al., 1993; Carvalho et al., 2005; Yore et al., 2014; Syed et al., 2018a). 9-PAHSA levels correlate highly with insulin sensitivity and are reduced in adipose tissue and serum of insulin-resistant humans. 9-PAHSA administration in mice lowered ambient glycemia and improved glucose tolerance while stimulating glucagon-like peptide-1 (GLP-1) and insulin secretion (Yore et al., 2014). 9-PAHSA also exhibited anti-inflammatory effects. For example, it decreased high fat diet (HFD)-induced adipose inflammation in obese, insulin-resistant mice and attenuated lipopolysaccharide (LPS)-induced dendritic cell activation and cytokine production *in vitro* (Moraes-Vieira et al., 2016). Notably, 9-PAHSA may also play a major role in mediating autophagy. We found 9-PAHSA treatment regulated autophagy-related pathway using iTRAQ approach in the study. Thus, the novel lipid 9-PAHSA opens up new opportunities of treatment for diabetes and cardiovascular complications.

In the study, we evaluated the effects of 9-PAHSA on DCVC and investigated the possible mechanisms underlying it. We found that 9-PAHSA ameliorated vascular calcification and myocardial dysfunction in db/db mice, possibly through promoting autophagic flux and reducing myocardial hypertrophy.

## Materials and Methods

### Human Samples Preparation

For the detection of serum 9-PAHSA levels, human blood samples were collected from 60 subjects including type 2 diabetes mellitus (T2DM) elderly patients (*n* = 30) and healthy elderly control subjects (control, *n* = 30). They were recruited randomly in the year of 2016 from the same group of the physical examination population of Huashan Hospital, Fudan University. The diagnosis of diabetes was according to the WHO criteria (fasting plasma glucose ≥7.0 mmol/L or 2 h plasma glucose ≥11.1 mmol/L during an oral glucose tolerance test). Subjects with type 1 diabetes, hypertension, severe psychological disorders, dementia, tumors, stroke, coronary heart disease, and acute or chronic inflammation were all excluded.

Data was collected on age, gender, body weight, height, waistline, hipline, blood pressure, serum lipid, blood glucose, and Hemoglobin A1C(HbA1C). Body mass index (BMI) was calculated based on body weight (kg) and height (m). Fasting blood samples were drawn after an overnight fast. Circulating HbA1C, glucose levels, and serum lipids were determined by the standard methods in the Huashan Hospital laboratory. Informed consent was provided by the participants. The experiments were performed in accordance with the Declaration of Helsinki of the World Medical Association, and approved by the ethics committee of Huashan Hospital, Fudan University (No. 2015-127).

### Animal Grouping and Intervention

Age matched male C57BL6/J and db/db mice were purchased from Nanjing University Biological Center (Nanjing, China), and housed in colony cages with free access to water and regular diet in a 12-h light/dark cycle and temperature-controlled environment. All the experimental procedures conformed to the NIH Guide for the Care and Use of Laboratory Animals. The Institutional Animal Care and Use Committee of Fudan University approved the experiments.

32-week-old mice were randomly assigned into groups as follows: ctrl + veh, db/db + veh, db/db+9-PAHSA (50 mg/kg per day) (n = 9 mice for each group). 9-PAHSA was given to mice by gavage once a day for 4 weeks. The veh groups were given with the same volume of vehicle (50% PEG400, 0.5% Tween-80, 49.5% H_2_O) at the corresponding time points.

### Serum Detection for 9-PAHSA

Serum samples were collected from human. Detection for serum 9-PAHSA levels in diabetic and non-diabetic human was performed by HPLC-MS/MS analysis. After centrifugation and pretreatment with phosphate-buffered saline, methanol and chloroform (1:1:1.5), the organic phase in the lower layer were collected and purified by using SPE column. Then, samples were separated and analyzed by using a Thermo Tsq Vantage HPLC-MS/MS (waters UHPLC T3) via multiple reaction monitoring in negative ionization mode (spray voltage 3,000 kV, atomization temperature 300°C, sheath gas pressure 40 psi, auxiliary gas pressure 15 psi, ion transmission tube temperature 350 °C). For gradient elution analysis, mobile phases contained (A) 5 mM ammonium acetate and (B) methanol consisted of 0.01% ammonium hydroxide. 9-PAHSA-d4 (10 ng/ml, Cayman) was used as internal standard.

### 9-PAHSA Synthesis

To investigate whether that supplement of 9-PAHSA benefit diabetic mice, the compound 9-PAHSA was synthesized by Prof. Jichang Xiao of Shanghai Institute of Organic Chemistry, Chinese Academy of Sciences with purity of >99% according to the previous paper (Yore et al., 2014). The characterization of synthesized 9-PAHSA was outlined in Figure 2.

### Detection for Fasting Glycemia

Fasting blood glucose (FBG) was tested three times in each mouse: prior to the administration of 9-PAHSA, and at 14 and 28 days after the administration of 9-PAHSA. In order to avoid glycemic fluctuations, mice were placed in a safe and quiet environment; this practice avoided provoking the animals. Venous blood was collected by cutting the tail vein of each mouse at 9:00 am every time. The oral glucose tolerance test (OGTT) was performed at 9:00 am after a single time intervention. Glucose levels were determined using Accu-Check active bands and a glucometer (Roche Diagnostics, Basel, Switzerland).

### Ultrasound Assessment of the Carotid Arterial Plaque

Mice were anesthetized by a mixed gas of oxygen and 2% isoflurane via nose cone. A heat lamp was used to keep mice warm during anesthesia. The neck of mice was shaved to reduce artifacts and then slightly hyperextended, after which each supine mouse was assessed via color doppler sonography using the Visual Sonics Vevo 770 high-resolution *in vivo* micro-imaging system with a micro-visualization scan head probe (RMV-707B) which had a focal length of 12.7 mm, a center frequency of 30 MHz, a −3 dB bandwidth of 15–45 MHz, an axial resolution of 55 μm, and a lateral resolution of 115 μm.

### Transthoracic Echocardiography

Transthoracic echocardiography was performed in sedated mice by using Vevo 770 high-resolution *in vivo* imaging system (30-MHz transducer; Visualsonics, Toronto, ON, Canada). Mice were anesthetized with 2% isoflurane. Basic hemodynamic parameters, such as left ventricular (LV) mass, left ventricular end-systolic internal diameter (LVIDs), left ventricular end-diastolic internal diameter (LVIDd), left ventricular end-systolic posterior wall thickness (LVPWs) and left ventricular end-diastolic posterior wall thickness (LVPWd) were measured by using M-mode.

### Detection for NT-Pro BNP

Abdominal aorta blood of mice was collected and detected for NT-pro BNP, by using ELISA kit (Sigma). Briefly, all samples were centrifuged, and plasma was tested according to the protocol of ELISA kit.

### Histopathology of Carotid Artery

After the animals were anesthetized and sacrificed, the left common carotid artery was obtained. Then, the samples were cut and stained with hematoxylin and eosin (HE) or alizarin red.

### Immunohistochemistry of Carotid Artery

The left common carotid artery was embedded in paraffin and sectioned at 5 um. Nonspecific binding was blocked with 10% normal rabbit serum. After incubation with polyclonal anti-vascular cell adhesion molecule-1 (VCAM-1) antibody (Santa Cruz Biotechnology, 4 ug/mL; 1:500) followed by anti-rabbit lgG secondary antibody, the slices were colored by diaminobenzidine to visualize positive immunoreactivity and counterstained with Hematoxylin.

### In-Solution Digestion/High pH RPLC

Cardiac protein samples of mice were reduced by 5 mM DTT at 56°C for 30 min and alkylated by 10 mM MMTS at room temperature for 30 min, then diluted with 50 mM ammonium bicarbonate until the urea concentration < 1 M. Lys-C was added at the mass ratio of 1:50 (enzyme: protein) for 3 h at 37°C. Then, trypsin was added at the mass ratio of 1:50 (enzyme: protein) for 12 h. For label-free quantification, the digested peptides were desalted using C18 column (Sep-Pak Vac C18, Waters Corporation), concentrated using SpeedVac, and then resuspended in 2% ACN with 0.1% FA. For iTRAQ samples, iTRAQ-8pLex labeling reagents (AB Sciex) were added to the peptide, which were incubated at room temperature for 120 min. The reaction was stopped by water, followed concentration using SpeedVac and desalts. The digested protein samples were fractionated by using high pH reversed phase liquid chromatography.

### Western Blot Analysis

Heart tissues (50–100 mg) were cut into small pieces and lysed with RIPA buffer (Roche, Switzerland), which consists of 50 mM Tris-HCl (pH 7.4), 150 mM NaCl, 0.5% sodium deoxycholate, 1% NP-40, 1% sodium dodecyl sulfate (SDS), and protease inhibitor cocktail, at 4 °C, then ruptured by homogenizer on ice. The supernatant was collected after centrifugation at 14,000×g for 30 min at 4°C. The protein concentration of the cell lysate was quantified by using the bicinchoninic acid assay (Beyotime Biotechnology, Beijing, China).

Cardiac proteins from mice were subjected to polyacrylamide gel electrophoresis and then transferred onto polyvinylidene difluoride membranes. The membranes were then probed with antibodies, including LC3-phosphatidyl ethanolamine conjugate (1:200), mTOR (1:1,000), GAPDH (1:2000) and PI3KIII (1:1,000) (all purchased from Cell signaling Technology, United States); BECN1 (1:500) and p-Akt (1:1,000) (all purchased from Santa Cruz); p62 (1:1,000), BAG3 (1:500) and HspB8 (1:500) (all purchased from Abcam, United Kingdom). Peroxidase activity was visualized with ECL (SantaCruz, United States). The bands were quantified using Quantity One.

### Transmission Electron Microscopy

Heart tissues of mice were perfused and fixed with 2.5% glutaraldehyde perfusate. Following the fixation and dehydration steps, the tissues were embedded in paraffin, sliced, and stained with 3% uranyl acetate and lead citrate. Cardiac ultrastructure was examined under transmission electron microscope (CM-120, Philips; Amsterdam, Netherlands). Six random fields of each slice were analyzed to calculate the relative area of autolysosomes and greases.

### Data and Statistical Analysis

All data are expressed as mean ± SEM and statistically analyzed by using the Graph Prism 7 software (GraphPad Software) and SPSS. ANOVA were used to evaluate the differences among multiple groups. Non-paired *t*-test was used to analyze two groups after homogeneity of variance test. Logistic regression was used to analyze the association between serum 9-PAHSA and T2DM. In the first model, we adjusted solely for age and gender. In Model 2 we further adjusted for BMI. We then added waistline, hipline in Model 3. In Model 4, fully-adjusted, model we adjusted for potential confounding factors, including SBP, DBP, TC, TG and LDL. *p* < 0.05 was considered statistically significant.

## Results

### Serum 9-PAHSA Levels Were Reduced in Elderly Diabetic Patients

**FIGURE 1:**
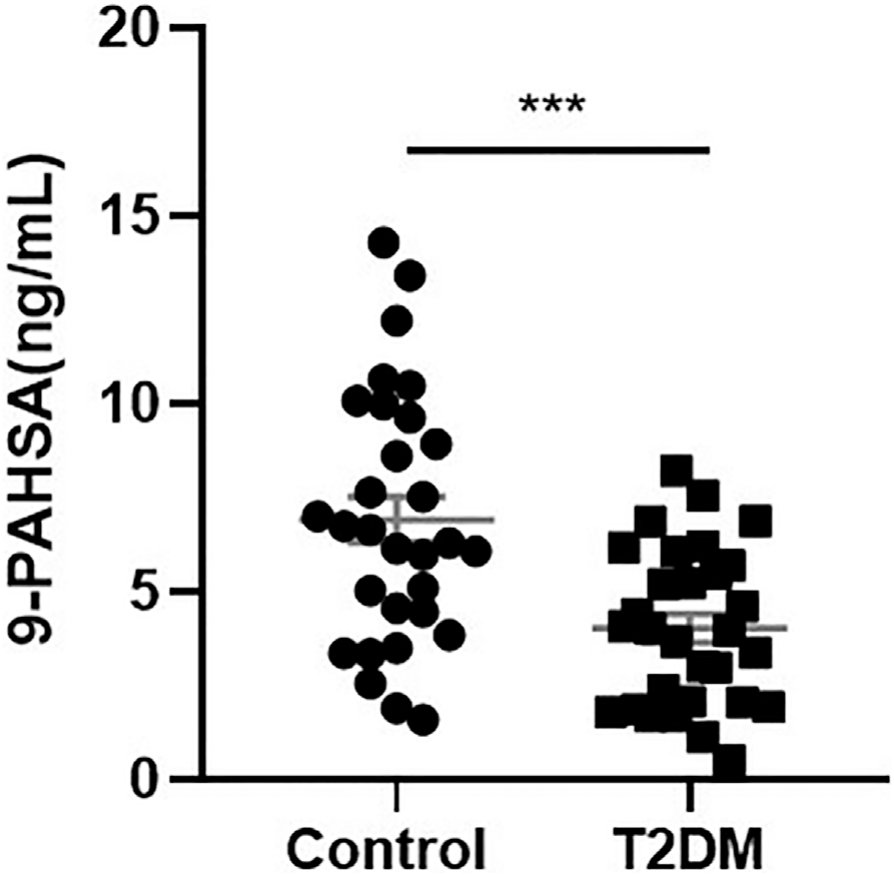
Serum 9-PAHSA Levels were decreased in diabetic patients. 9-PAHSA levels were detected by using UHPLC-MS/MS. ***p < 0.001, n = 30 for control, n = 30 for T2DM. Data are presented as mean ± SE.

To determine if 9-PAHSA was regulated in diabetic state, we firstly detected serum 9-PAHSA levels from type 2 diabetic humans using HPLC-MS/MS analysis. The results showed that serum 9-PAHSA was reduced in diabetic patients compared to the nondiabetic humans (Figure 1). The baseline characteristics of the T2DM and control groups are listed (Table 1). Briefly, T2DM patients have significantly higher blood glucose and HbA1C levels compared to nondiabetic humans. There were no differences in age, gender, BMI, blood pressure, waistline, hipline, and serum lipids.

**TABLE 1:** Physiological parameters of the study population in healthy control and T2DM group (n = 30 for each group, mean ± SE).

Variables	Control (n = 30)	T2DM (n = 30)	*p*-value^a^
Age (years)	74.5 ± 6.7	74.5 ± 6.6	1.000
Male (n)	18	16	0.998
Female (n)	12	14	0.998
Body weight (kg)	64.4 ± 5.8	63.6 ± 6.0	0.764
Waistline (cm)	81.6 ± 6.0	85.5 ± 7.7	0.223
Hipline (cm)	94.5 ± 4.3	97.9 ± 4.3	0.097
BMI (kg/m^2^)	22.6 ± 1.5	22.6 ± 1.6	0.976
SBP (mmHg)	137.6 ± 17.2	138.1 ± 11.6	0.905
DBP (mmHg)	75.3 ± 8.0	73.8 ± 9.7	0.570
BG (mmol/L)	5.3 ± 0.5	6.7 ± 1.1	0.001^***^
HbA1C (%)	5.6 ± 0.3	6.8 ± 0.9	0.001^***^
TC (mmol/L)	4.8 ± 0.8	4.6 ± 1.0	0.303
LDL (mmol/L)	2.8 ± 0.7	2.7 ± 0.9	0.723
TG (mmol/L)	1.1 ± 0.4	1.3 ± 0.9	0.354

a *p*-value for comparisons between control and T2DM group by an independent samples *t*-test or Chi-square test. ***p < 0.001. BMI—body mass index; SBP—systolic blood pressure; DBP—diastolic blood pressure; BG—blood glucose; HbA1C—glycosylated hemoglobin; TC—total cholesterol; LDL—low-density lipoprotein; TG—triglyceride.

Serum 9-PAHSA was a protective factor for T2DM (Table 2, OR 0.71, 95%CI: 0.58, 0.87). After adjusting for age and gender, 9-PAHSA was still a protective factor for T2DM (Model 1, OR 0.69, 95%CI: 0.53, 0.89). Further adjustment for BMI, waistline and hipline, only minimally changed this association (Model 2 and 3). Moreover, the estimated association between serum 9-PAHSA and T2DM also appeared slightly changed when we adjusted for SBP, DBP, TC and LDL (Model 4, OR 0.50, 95%CI: 0.30, 0.83).

**TABLE 2:** Multiple logistic regression analysis of correlation between serum 9-PAHSA and T2DM.

Group	Unadjusted		Model 1		Model 2		Model 3		Model 4	
	OR	95%-CI	OR	95%-CI	OR	95%-CI	OR	95%-CI	OR	95%-CI
Control	…	…	…	…	…	…	…	…	…	…
T2DM	0.71	(0.58,0.87)	0.69	(0.53, 0.89)	0.68	(0.53, 0.89)	0.55	(0.34, 0.89)	0.50	(0.30, 0.83)

OR = odds ratio for type 2 diabetes mellitus; CI = confidence interval.

Model 1: adjusted for age and gender.

Model 2: adjusted for age, gender and BMI.

Model 3: adjusted for age, gender, BMI, waistline and hipline.

Model 4: adjusted for age, gender, BMI, waistline, hipline, SBP, DBP, TC and LDL.

### 9-PAHSA Was Successfully Synthesized and Characterized

**FIGURE 2:**
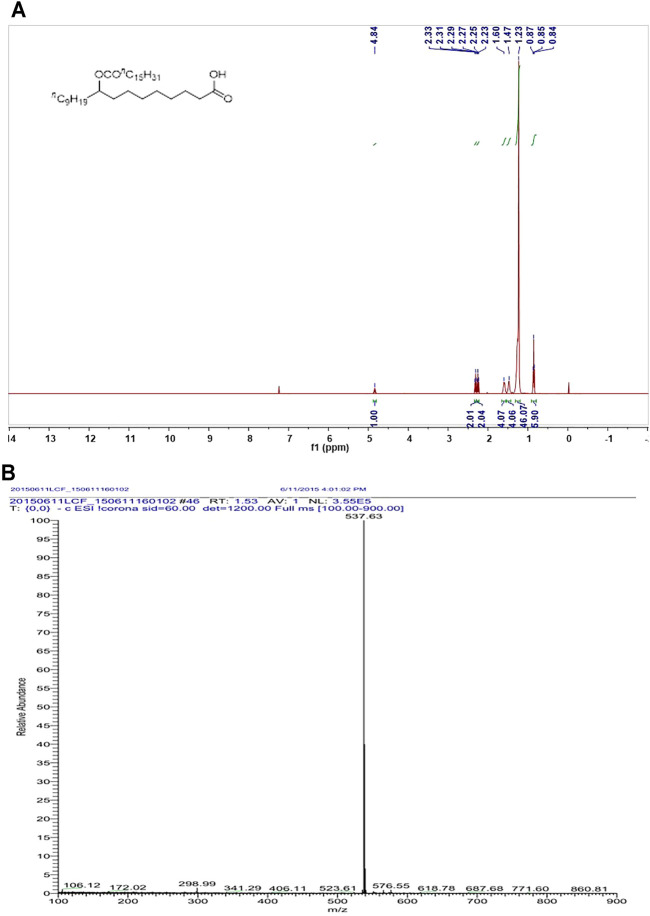
The synthesis and characterization of 9-PAHSA. **(A)** ^1^H NMR of 9-PAHSA. **(B)** ESI-MS spectrometry of 9-PAHSA.

Since 9-PAHSA levels were decreased in diabetic state, we synthesized 9-PAHSA compound for the following exogenous supplement experiments. Detailed procedure for synthesize of 9-PAHSA was available in Supplementary Materials (S1). 9-PAHSA was fully characterized by ^1^H NMR (Figure 2A) and electrospray ionization-mass spectrometry (ESI-MS) spectrometry (Figure 2B). These data demonstrated that 9-PAHSA was successfully and correctly synthesized.

### 9-PAHSA Administration Lowered Glycemia in Db/Db Mice

**FIGURE 3:**
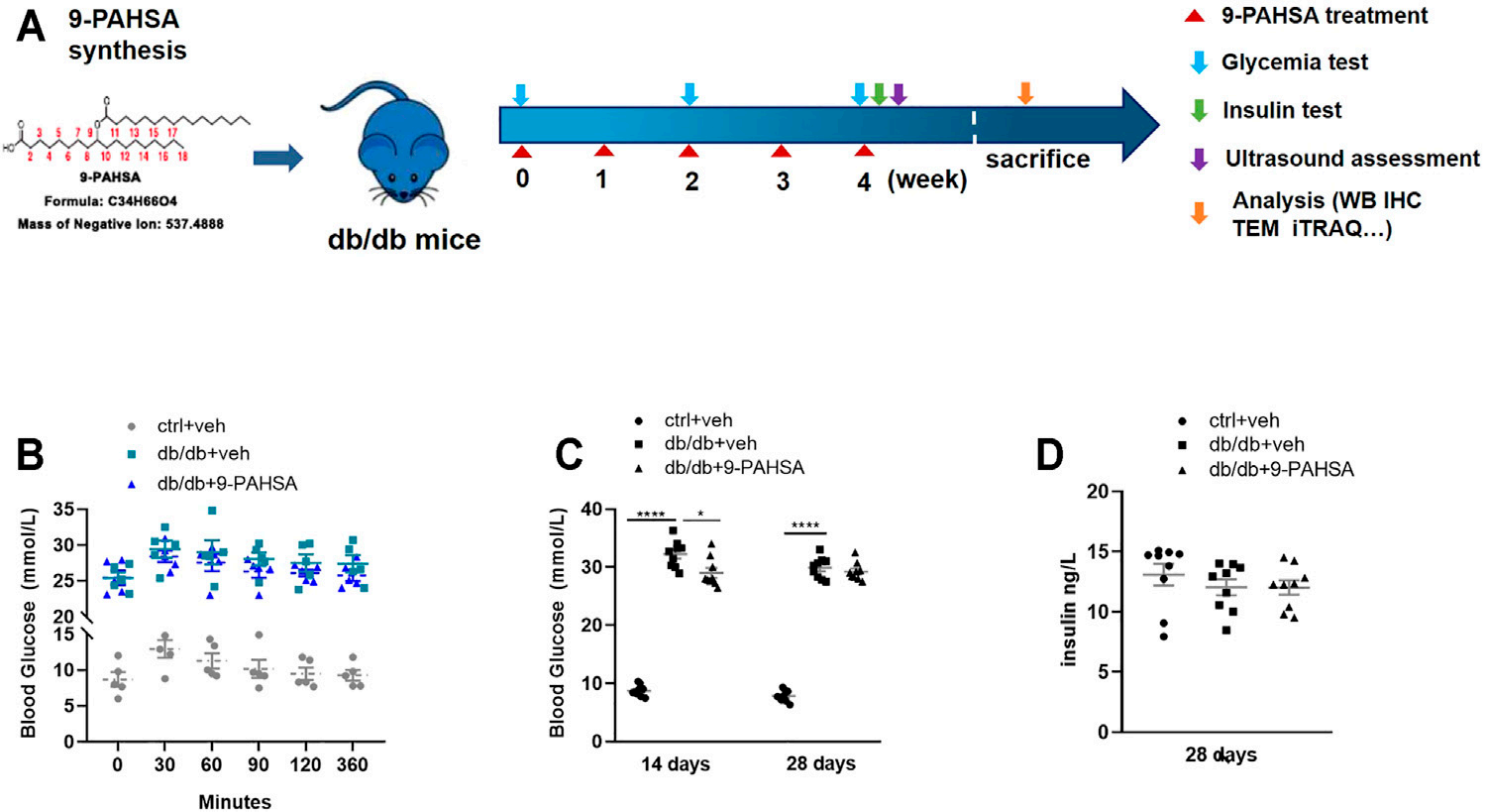
Effects of 9-PAHSA administration on glucose homeostasis in db/db mice. **(A)** The experimental design. **(B)** Oral glucose tolerance test (OGTT) was performed 30 min after 9-PAHSA administration. Serum glucose was detected right after the administration and at the 30, 60, 90, 120, 360 min thereafter. n = 5 for each group. **(C)** Glycemia was measured after 2 weeks and 4 weeks administration of 9-PAHSA. **(D)** Insulin was measured after 4 weeks administration of 9-PAHSA. *p < 0.05, ****p < 0.0001. n = 9 for each group (C, D). Data are presented as mean ± SE.

We then investigated whether administration of 9-PAHSA could improve glucose homeostasis and diabetic associated vascular diseases (Figure 3A). 9-PAHSA slightly improved glucose tolerance with reduced area under the glucose excursion curve after acutely administration (Figure 3B), while remarkably induced glucose-lowering compared with vehicle treatment in db/db mice after 2 weeks administration (Figure 3C). There was no difference in plasma insulin levels in9-PAHSA-treated db/db mice compared to vehicle-treated db/db mice after 4 weeks administration (Figure 3D). These results indicated that 9-PAHSA administration might partly reduce blood glucose levels and temporarily enhance insulin sensitivity.

### 9-PAHSA Attenuated Carotid Arterial Atherosclerosis in Db/Db Mice

**FIGURE 4:**
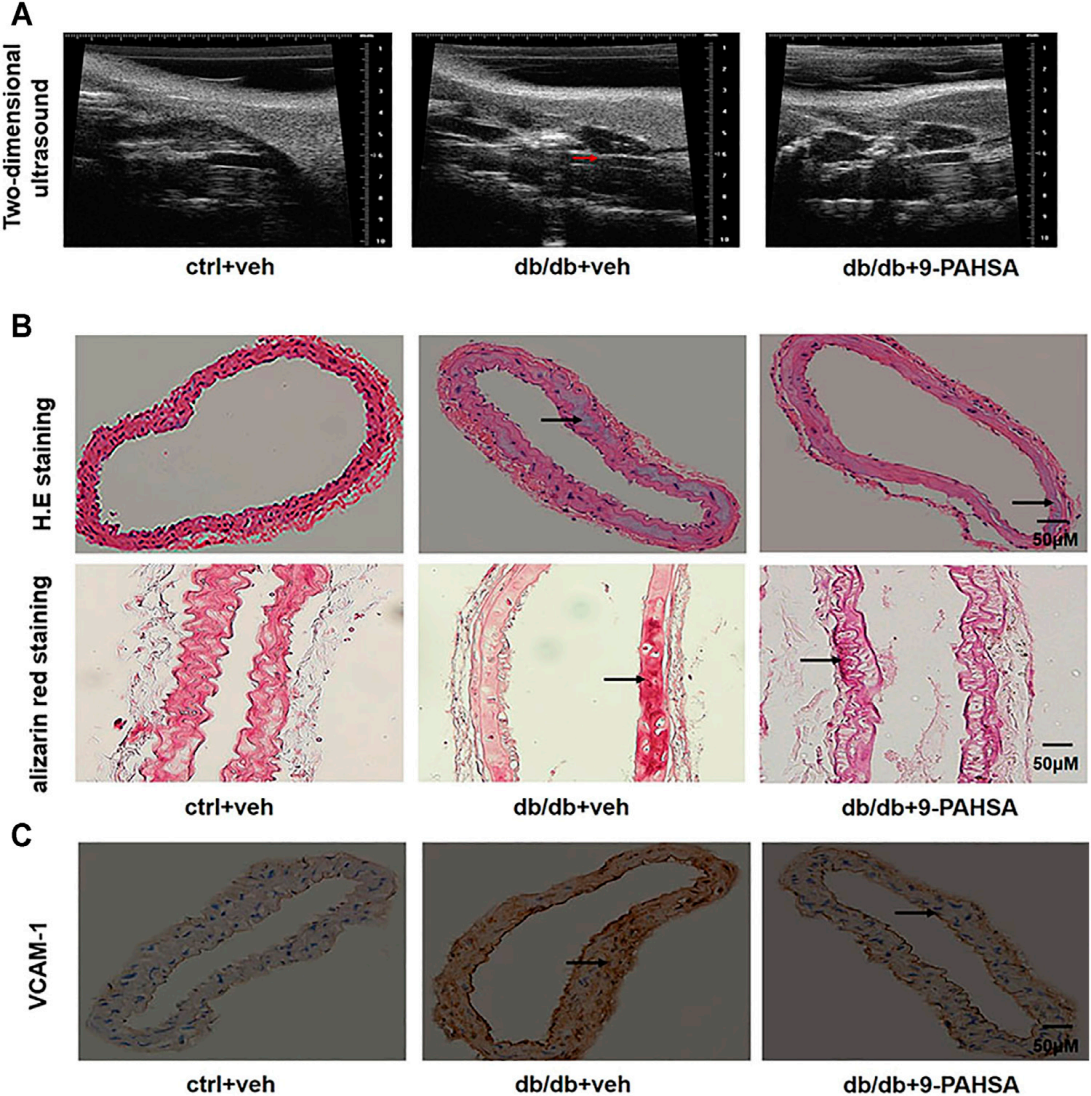
Effects of 9-PAHSA on carotid arterial atherosclerosis in db/db mice. **(A)** Two-dimensional ultrasound assessments of arterial wall plaque in the left common carotid artery. Arrows represent arterial wall plaque. **(B)** H.E and alizarin red staining cross-sections of the left common carotid arteries to assess vascular calcification. Arrows represent vascular calcification. **(C)** Immunohistochemistry showed VCAM-1 expression in the left common carotid arteries. n = 6 for each group. Arrows represent VCAM-1 expression.

In order to investigate the effect of 9-PAHSA on vascular atherosclerosis in db/db mice, we conducted two-dimensional ultrasound to observe the left common carotid arterial plaques. The carotid arterial plaques were notably observed in db/db mice but remarkably attenuated in 9-PAHSA-treated db/db mice (Figure 4A). H and E staining showed that each layer of the vascular wall was clear and orderly arranged, with no calcification in the control mice. In db/db mice, obvious calcification appeared. There was less calcification in 9-PAHSA-treated db/db mice compared to the vehicle-treated db/db mice (Figure 4B). Similarly, vascular alizarin red staining further verified the results (Figure 4B).

VCAM-1is a member of the transmembrane immunoglobulin (Ig) superfamily. It plays an important role in the initiation and progression of atherosclerosis (Ponnuswamy et al., 2012). In this study, more VCAM-1 was observed in media smooth muscle cells of the left common carotid artery in db/db mice compared to control mice. 9-PAHSA treatment notably decreased endothelial VCAM-1 distribution in db/db mice (Figure 4C). These results suggested that 9-PAHSA could attenuate the atherosclerosis of carotid vascular in db/db mice.

### 9-PAHSA Ameliorated Myocardial Hypertrophy and Dysfunction in Db/Db Mice

**FIGURE 5:**
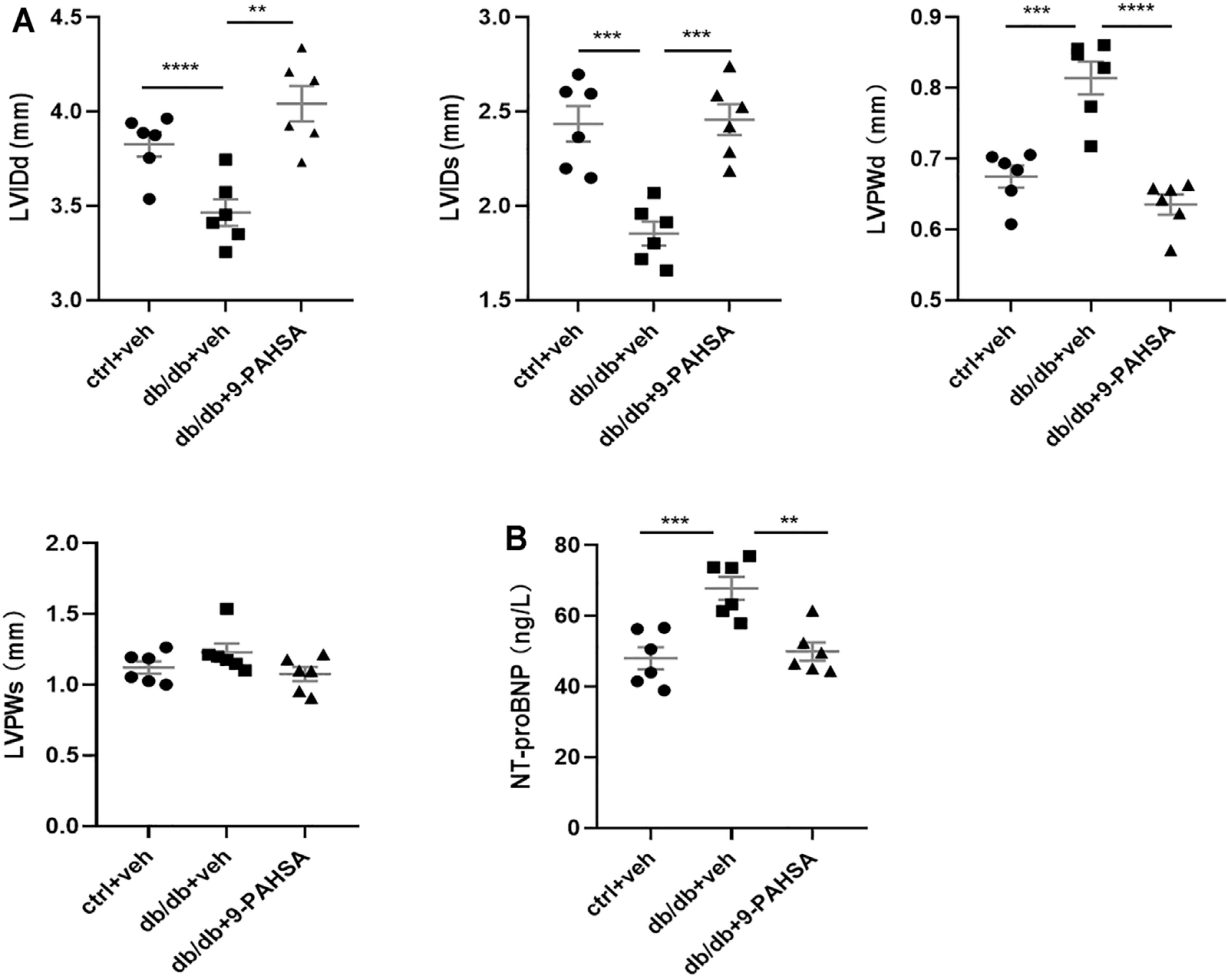
Effects of 9-PAHSA on myocardial hypertrophy and LV function in db/db mice. Cardiac structure **(A)** was assessed by transthoracic echocardiography **(B)** The contents of serum NT-proBNP were detected by ELISA kit. **p < 0.01, ***p < 0.001, ****p < 0.0001, n = 6 for each group. Data are presented as mean ± SE. Left ventricular internal diameter end diastole (LVIDd), left ventricular internal diameter end systole (LVIDs), left ventricular posterior wall end diastole (LVPWd), left ventricular posterior wall end systole (LVPWs).

In view of the above improvement of carotid atherosclerosis and calcification in diabetic mice by 9-PAHSA, we further investigated whether 9-PAHSA had a positive effect on cardiovascular function in diabetic mice by performing transthoracic echocardiography after 4 weeks administration of 9-PAHSA in db/db mice. Compared with the control mice, db/db mice showed the decreased end-diastolic and end-systolicleft ventricular internal diameter (LVIDd and LVIDs), but the increased left ventricular posterior wall depth at the end-diastole and end-systole (LVPWd and LVPWs), indicating that diabetes induced myocardial hypertrophy in db/db mice. 9-PAHSA administration significantly increased LVIDd and LVIDs, while decreased LVPWd and LVPWs in db/db mice (Figure 5A), suggesting that 9-PAHSA treatment ameliorated diabetes-induced myocardial hypertrophy.

The level of serum N-terminal prob-type natriuretic peptide (NT-proBNP) is a critical index to evaluate cardiac function. It is a widely used biomarker in diagnosing cardiac insufficiency. Our results found that the level of NT-proBNP was significantly higher in db/db mice compared to control mice (Figure 5B), indicating that db/db mice might suffer from chronic cardiac insufficiency. 9-PAHSA reduced NT-proBNP level (Figure 5B), as suggested to be protective against cardiac dysfunction. Altogether, these results demonstrated that db/db mice had slowly impaired cardiac functions with lesser left ventricle internal diameter and thicker left ventricle wall. Continuous 9-PAHSA administration could improve the cardiac functional and structural profiles in db/db mice.

### 9-PAHSA Enhanced Cardiac Autophagy in Db/Db Mice

To explore the underlying mechanisms of 9-PAHSA protection against diabetic cardiomyopathy, we analyzed the effect of 9-PAHSA on the myocardial proteomics of mice using the iTRAQ approach. A total of 432 differentially expressed proteins were identified among the control mice, db/db mice and 9-PAHSA-treated db/db mice. Among them, 82 proteins were regulated by 9-PAHSA treatment, which were involved in lipid metabolism, mitochondrial functions, cardiomyopathy-related, and autophagy-related pathways. The regulation of all these proteins may collectively contributed to the biological effect of 9-PAHSA on the heart of db/db mice.

**FIGURE 6:**
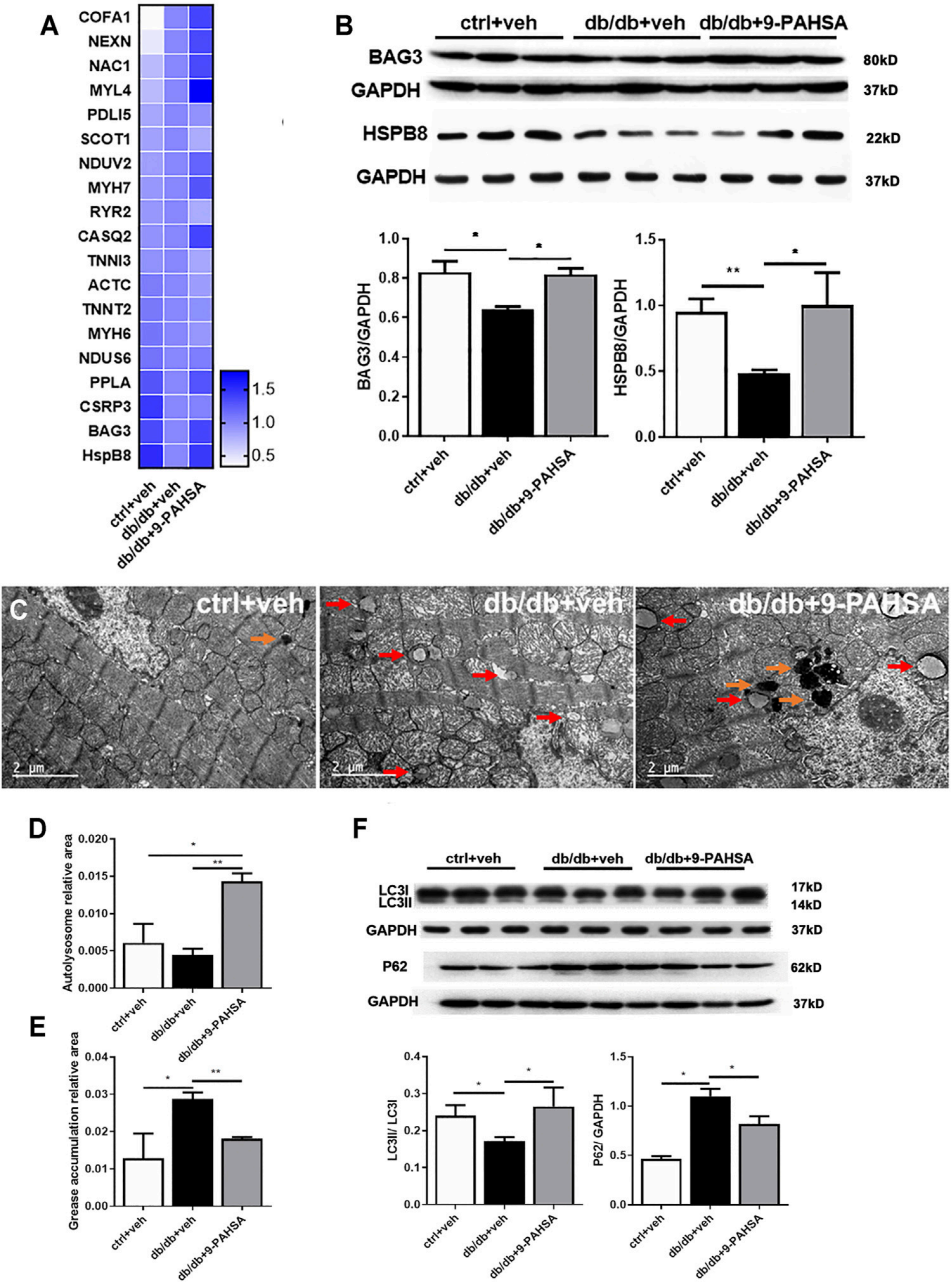
Effects of 9-PAHSA treatment on cardiac autophagy. **(A)** Represetative altered proteins after 9-PAHSA treatment by using iTRAQ analysis. **(B)** Western blotting analysis detected the expression levels of BAG3 and HSPB8. **(C)** Representative cardiomyocyte electron micrographs of ctrl + veh, db/db + veh and db/db+9-PAHSA mice. 9-PAHSA intervention increased the number of autolysosomes **(orange arrow)** and reduced the grease aggregation **(red arrow)** in db/db cardiomyocytes. **(D and E)** Statistical column graphs representing the ratio of autolysosome area **(D)** and grease aggregates area **(E)**. **(F)** Representative western blotting images and statistical columns of cardiac LC3 and P62 protein levels, which showed that cardiac autophagy levels were enhanced under 9-PAHSA administration in db/db mice. *p < 0.05, **p < 0.01, n = 3 mice for each group. Data are presented as mean ± SE.

Nevertheless, to gain a better understanding of the physiological roles of 9-PAHSA on diabetic cardiomyopathy, we further confirmed the changes of two proteins among the above 82 proteins list. They were Bcl2-associated athanogene 3 (BAG3) and heat shock protein beta-8 (HSPB8), which were upregulated after 9-PAHSA intervention by iTRAQ analysis (Figure 6A), and further confirmed by western blotting analysis (Figure 6B). BAG3/HSPB8 complex is involved in enhancing myocardial autophagy. The impairment of autophagy contributes to the progress of diabetes-induced cardiac abnormalities (Bartlett et al., 2017). It is hypothesized that 9-PAHSA could improve diabetic cardiomyopathy in db/db mice by increasing cardiac autophagy. To determine whether 9-PAHSA mediated cardiac autophagy in db/db mice, we examined the morphological images by using electron microscopy. In control mice, the cardiomyocytes were regularly arranged, with few autophagosomes and lysosomes in the cytosol. In contrast, small decreased autolysosomes but apparent increased greases were observed in the myocardial cells of the db/db mice. However, 9-PAHSA treatment significantly increased autolysosomes, while dramatically decreased greases in the myocardial cells of the db/db mice (Figures 6C–E).

In addition, results of western blotting analysis showed the decreased ratio of LC3II/LC3I and increased P62 protein level in db/db mice compared to control mice. However, 9-PAHSA treatment partly reversed the expression of these proteins in db/db mice (Figure 6F). These results suggested that 9-PAHSA enhanced cardiac autophagy in db/db mice.

### 9-PAHSA Increased Cardiac Autophagy Through p-AKT/mTOR/PI3KIII-BECN-1 Pathway in Db/Db Mice

**FIGURE 7:**
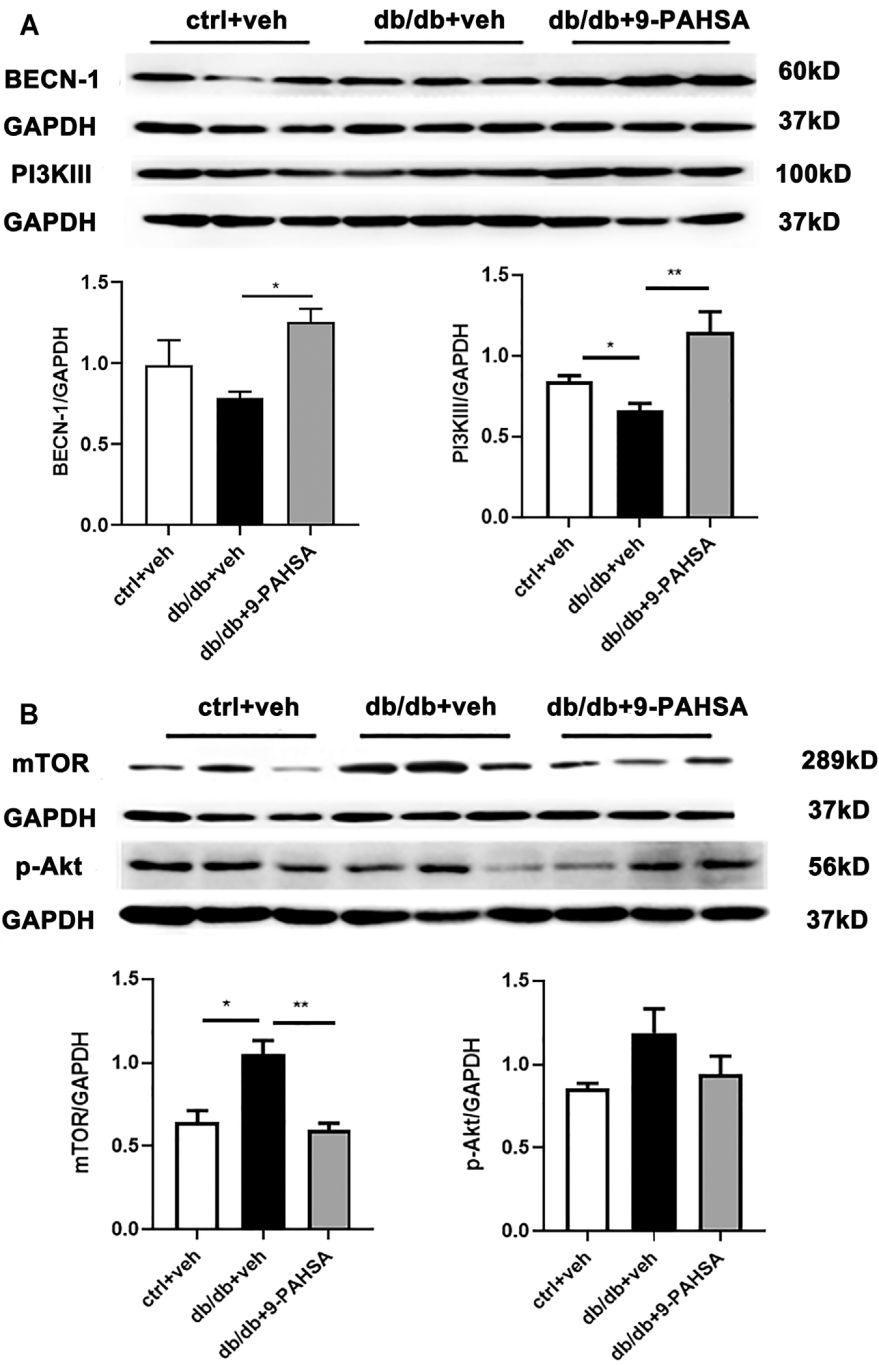
Effects of 9-PAHSA on the expression of autophagy-related proteins in cardiomyocytes of db/db mice. Western blot analysis was used to detect protein levels of cardiac BECN-1, PI3K III, mTOR, p-Ak after 9-PAHSA treatment in db/db mice. *p < 0.05, **p < 0.01, n = 3 for each group. Data are presented as mean ± SE.

In order to elucidate the possible autophagic signaling pathways involved in the effect of 9-PAHSA, we detected the cardiac levels of several proteins which related to autophagic pathways. Our results showed that cardiac BECN1 and PI3KIII levels were reduced in db/db mice while significantly increased after 9-PAHSA treatment (Figure 7A). Meanwhile, the expression of mTOR in the myocardium increased in db/db mice, while reduced in 9-PAHSA-treated db/db mice (Figure 7B). Besides, 9-PAHSA exert mild tendency of reduction in p-Akt level when compared with db/db mice (Figure 7B). These results suggested that 9-PAHSA treatment increased cardiac autophagy possibly via up-regulation of PI3KIII and BECN1, and down-regulation of mTOR and p-Akt in diabetic myocardium.

## Discussion

In this study, we demonstrated a new role of 9-PAHSA, that is, continued administration of 9-PAHSA alleviates cardiovascular complications by promoting autophagic flux and reducing myocardial hypertrophy in db/db mice. As one of the endogenous metabolic products of palmitic acid, 9-PAHSA has been reported to reduce hyperglycemia (Yore et al., 2014). In our study, we found that 9-PAHSA levels are reduced in T2DM. Therefore, it is supposed that the exogenous supplementation of 9-PAHSA might be an effective means for the therapy of T2DM. A single oral dose of 9-PAHSA improves glucose tolerance in insulin resistant mice (Yore et al., 2014). Our data showed that 9-PAHSA played a role in glucose-lowering in db/db mice after 2 weeks administration. But this action disappeared after 4 weeks administration. Unconsistent with it, it is reported that chronic PAHSA treatment in 15-week-old HFD mice improved insulin sensitivity and glucose tolerance and these effects were sustained for at least 4.5 months (Syed et al., 2018b). Such discrepancy might due to the age of the mice we used are much closer to the senile period of diabetic mice, because the occurrence of T2DM is highly associated with aging (Saeedi et al., 2019). Although our results seem to be more similar to the real clinic cases, the hypoglycemic effect of 9-PAHSA in elderly patients need further investigation.

Cardiovascular complication remains to be the principal cause of death and disability among patients with diabetes mellitus. Multi-factorial risk is highly associated with diabetic associated cardiac disorders, such as hypertension, hyperglycemia, hyperlipemia and atherosclerosis (Bornfeldt, 20132014; Low Wang et al., 2016). Therefore, it is not adequate to modulate cardiovascular disorder by therapeutic strategies focusing solely on optimal glycemic control. Developing drugs that focus on cardiovascular management, but not simply on glycemic control is of the utmost importance for DCVC patients. As a novel endogenous fatty acid, we demonstrated new roles of 9-PAHSA in relieving atherosclerosis and cardiac failure in aging diabetic mice. It has been extensively documented that certain fatty acids play beneficial roles in cardiovascular diseases (Sokola-Wysoczanska et al., 2018). Polyunsaturated fatty acids (PUFAs) belong to fatty acids family and activate the G-protein coupled receptor (GPR) 120/free fatty acid receptor (FFAR) 4 (GPR120/FFAR4) signaling (Harper and Jacobson, 2001; Oh et al., 2010). The supplement of omega-3 PUFAs could improve cardiac structures (Harper and Jacobson, 2001). Similar to PUFA, 9-PAHSA is the ligand of GPR120 and also exerts anti-diabetic and cardiac protective effects.

Autophagy plays an important role in the maintenance of normal heart function. Autophagy could be triggered by metabolic clues. Accumulating evidence suggested that autophagy was impaired in the heart with insulin resistance and T2DM (Xu et al., 2013; Kanamori et al., 2015; Munasinghe et al., 2016). However, whether the suppression of cardiac autophagy has beneficial or detrimental functional consequences in T2DM is largely unknown. Studies showed that inhibition of autophagy lead to ventricular hypertrophy (Kenessey and Ojamaa, 2016; Zeng et al., 2017; Zhang et al., 2017). In contrast, it is also reported that the reduced cardiac autophagy in diabetic mice promoted the progression of cardiac aging (Eisenberg et al., 2016; Shirakabe et al., 2016). Studies involving autophagy assessment in the hearts of diabetic mice have been controversial. This may be due in part to the different mouse models using in studies and metabolic complexity of these conditions. Our study used the db/db mouse, a genetic model of T2DM. Consistently, we found that cardiac autophagic level is reduced in db/db mice, as evidenced by the decreased expression of cardiac HSPB8, BECN1 and PI3KIII proteins and enhanced level of cardiac mTOR protein. Moreover, we showed that enhanced autophagy regulated by 9-PAHSA alleviated diabetic cardiomyopathy in db/db mice.

Akt/mTOR signaling activation inhibits cell autophagy. In the study, we found that the pathway was elevated in hearts of db/db mice. mTOR inhibits autophagy through suppressing the activation of PI3KIII/BECN1 complex (Kim et al., 2011; Heras-Sandoval et al., 2014). Besides, the reduced levels of cardiac BAG3 and HSPB8 were identified in db/db mice. BAG3 is one of a family of co-chaperones characterized by a C-terminal BAG domain that binds the HSP70/HSPA ATPase domain to regulate the fate of HSP70 substrates. BAG3 functions as a chaperone interacting with HspB8 and targets misfolded proteins to macroautophagy. Studies have reported that BAG3/HSPB8 complex is involved in autophagic signaling (Carra et al., 2008; Li et al., 2017). BAG3 would promote the sequestration and targeting of HSP70/HSC70-associated protein aggregates to the aggresome, a perinuclear compartment with high autophagic activity. While overexpression of HSPB8 can stimulate autophagy in a BAG3-dependent manner, it seems to be dispensable for the function of BAG3 in the aggresome-autophagy pathways during proteotoxic stress (Fuchs et al., 2015). It is further demonstrated that autophagic flux was impaired in the heart of db/db mice. It is worth noting that 9-PAHSA treatment down-regulated Akt/mTOR and activated PI3KIII/BECN1 complex in diabetic myocardium, suggesting that 9-PAHSA could promote cardiac autophagy.

**FIGURE 8:**
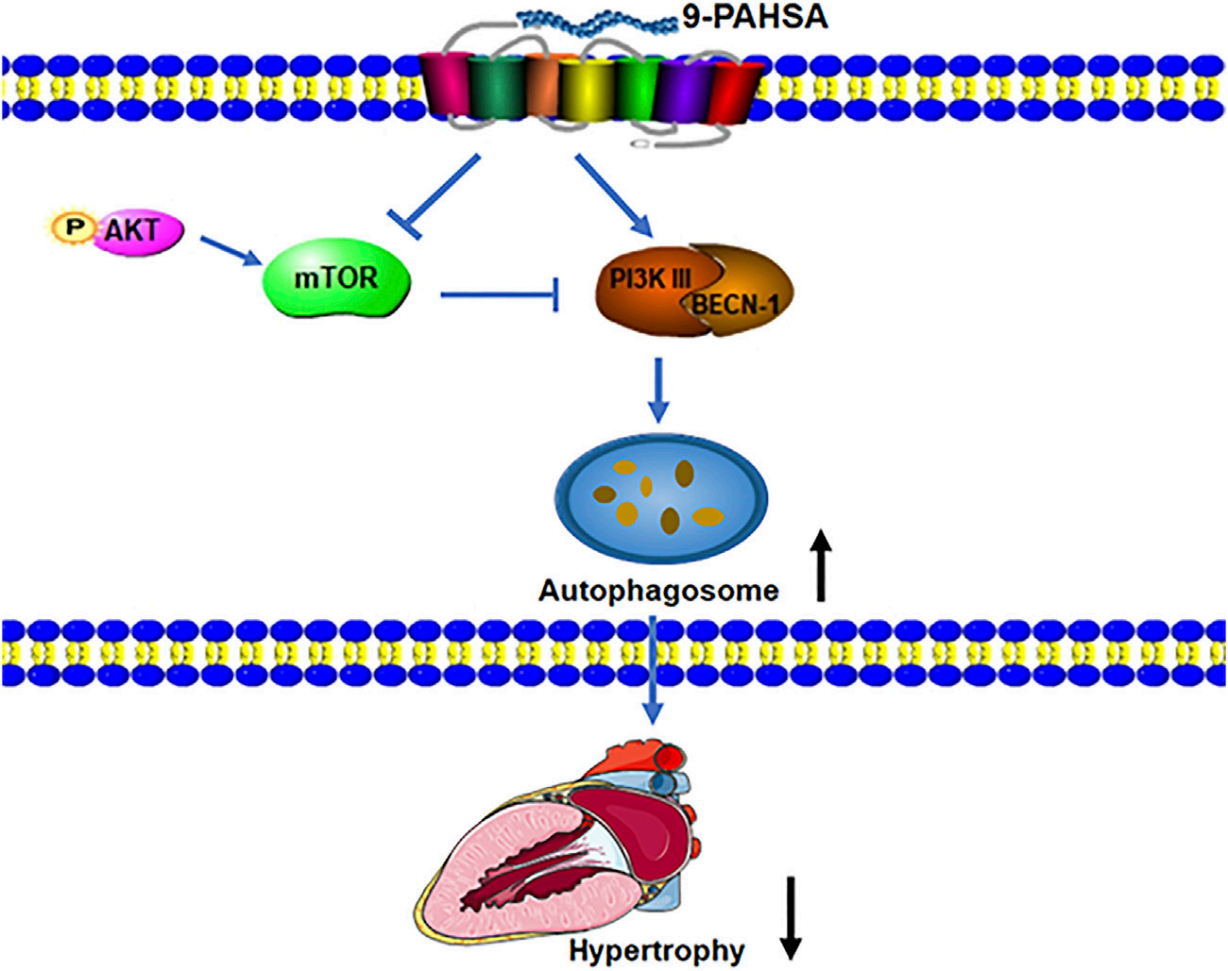
9-PAHSA increased autophagy to ameliorate cardiac hypertrophy via up-regulation of PI3KIII and BECN1 and down-regulation of mTOR and p-Akt in diabetic myocardium.

Taken together, our results demonstrated that continued administration of 9-PAHSA alleviated diabetic cardiomyopathy in db/db mice.9-PAHSA treatment increased cardiac autophagy possibly via up-regulation of PI3KIII and BECN1 and down-regulation of mTOR and p-Akt in diabetic myocardium (Figure 8). The exogenous supplementation of 9-PAHSA might be an effective strategy for the T2DM-related cardiomyopathy.

## Data Availability

The data that support the findings of this study are available from the corresponding author upon reasonable request.

## References

[B1] Bartlett J. J. Trivedi P. C. Pulinilkunnil T. (2017). Autophagic Dysregulation in Doxorubicin Cardiomyopathy. J. Mol. Cel Cardiol 104, 1–8. 10.1016/j.yjmcc.2017.01.007 28108310

[B2] Bornfeldt K. E. (20132014). 2013 Russell Ross Memorial Lecture in Vascular Biology: Cellular and Molecular Mechanisms of Diabetes Mellitus-Accelerated Atherosclerosis. Arterioscler Thromb. Vasc. Biol. 34, 705–714. 10.1161/ATVBAHA.113.301928 PMC3967130 24665124

[B3] Carra S. Seguin S. J. Landry J. (2008). HspB8 and Bag3: a New Chaperone Complex Targeting Misfolded Proteins to Macroautophagy. Autophagy 4, 237–239. 10.4161/auto.5407 18094623

[B4] Carvalho E. Kotani K. Peroni O. D. Kahn B. B. (2005). Adipose-specific Overexpression of GLUT4 Reverses Insulin Resistance and Diabetes in Mice Lacking GLUT4 Selectively in Muscle. Am. J. Physiol. Endocrinol. Metab. 289, E551–E561. 10.1152/ajpendo.00116.2005 15928024

[B5] Eisenberg T. Abdellatif M. Schroeder S. Primessnig U. Stekovic S. Pendl T. (2016). Cardioprotection and Lifespan Extension by the Natural Polyamine Spermidine. Nat. Med. 22, 1428–1438. 10.1038/nm.4222 27841876 PMC5806691

[B6] Fuchs M. Luthold C. Guilbert S. M. Varlet A. A. Lambert H. Jetté A. (2015). A Role for the Chaperone Complex BAG3-HSPB8 in Actin Dynamics, Spindle Orientation and Proper Chromosome Segregation during Mitosis. Plos Genet. 11, e1005582. 10.1371/journal.pgen.1005582 26496431 PMC4619738

[B7] Harper C. R. Jacobson T. A. (2001). The Fats of Life: the Role of omega-3 Fatty Acids in the Prevention of Coronary Heart Disease. Arch. Intern. Med. 161, 2185–2192. 10.1001/archinte.161.18.2185 11575974

[B8] Heras-Sandoval D. Pérez-Rojas J. M. Hernández-Damián J. Pedraza-Chaverri J. (2014). The Role of PI3K/AKT/mTOR Pathway in the Modulation of Autophagy and the Clearance of Protein Aggregates in Neurodegeneration. Cell Signal 26, 2694–2701. 10.1016/j.cellsig.2014.08.019 25173700

[B9] Kanamori H. Takemura G. Goto K. Tsujimoto A. Mikami A. Ogino A. (2015). Autophagic Adaptations in Diabetic Cardiomyopathy Differ between Type 1 and Type 2 Diabetes. Autophagy 11, 1146–1160. 10.1080/15548627.2015.1051295 26042865 PMC4590644

[B10] Kenessey A. Ojamaa K. (2016). Thyroid Hormone Stimulates Protein Synthesis in the Cardiomyocyte by Activating the Akt-mTOR and p70S6K Pathways. J. Biol. Chem. 281, 20666–20672. 10.1074/jbc.M512671200 16717100

[B11] Kim J. Kundu M. Viollet B. Guan K. L. (2011). AMPK and mTOR Regulate Autophagy through Direct Phosphorylation of Ulk1. Nat. Cel Biol 13, 132–141. 10.1038/ncb2152 PMC3987946 21258367

[B12] Kovacic J. C. Castellano J. M. Farkouh M. E. Fuster V. (2014). The Relationships between Cardiovascular Disease and Diabetes: Focus on Pathogenesis. Endocrinol. Metab. Clin. North. Am. 43, 41–57. 10.1016/j.ecl.2013.09.007 24582091

[B13] Li X. C. Hu Q. K. Chen L. Liu S. Y. Su S. Tao H. (2017). HSPB8 Promotes the Fusion of Autophagosome and Lysosome during Autophagy in Diabetic Neurons. Int. J. Med. Sci. 14, 1335–1341. 10.7150/ijms.20653 29200947 PMC5707750

[B14] Low Wang C. C. Hess C. N. Hiatt W. R. Goldfine A. B. (2016). Clinical Update: Cardiovascular Disease in Diabetes Mellitus: Atherosclerotic Cardiovascular Disease and Heart Failure in Type 2 Diabetes Mellitus - Mechanisms, Management, and Clinical Considerations. Circulation 133, 2459–2502. 10.1161/CIRCULATIONAHA.116.022194 27297342 PMC4910510

[B15] Luo G. Jian Z. Zhu Y. Zhu Y. Chen B. Ma R. (2019). Sirt1 Promotes Autophagy and Inhibits Apoptosis to Protect Cardiomyocytes from Hypoxic Stress. Int. J. Mol. Med. 43, 2033–2043. 10.3892/ijmm.2019.4125 30864731 PMC6443335

[B16] Moraes-Vieira P. M. Saghatelian A. Kahn B. B. (2016). GLUT4 Expression in Adipocytes Regulates De Novo Lipogenesis and Levels of a Novel Class of Lipids with Antidiabetic and Anti-inflammatory Effects. Diabetes 65, 1808–1815. 10.2337/db16-0221 27288004 PMC4915575

[B17] Munasinghe P. E. Riu F. Dixit P. Edamatsu M. Saxena P. Hamer N. S. (2016). Type-2 Diabetes Increases Autophagy in the Human Heart through Promotion of Beclin-1 Mediated Pathway. Int. J. Cardiol. 202, 13–20. 10.1016/j.ijcard.2015.08.111 26386349

[B18] Oh D. Y. Talukdar S. Bae E. J. Imamura T. Morinaga H. Fan W. (2010). GPR120 Is an omega-3 Fatty Acid Receptor Mediating Potent Anti-inflammatory and Insulin-Sensitizing Effects. Cell 142, 687–698. 10.1016/j.cell.2010.07.041 20813258 PMC2956412

[B19] Ponnuswamy P. Schröttle A. Ostermeier E. Grüner S. Huang P. L. Ertl G. (2012). eNOS Protects from Atherosclerosis Despite Relevant Superoxide Production by the Enzyme in apoE Mice. PLoS One 7, e30193. 10.1371/journal.pone.0030193 22291917 PMC3264598

[B20] Saeedi P. Petersohn I. Salpea P. Malanda B. Karuranga S. Unwin N. , Global and Regional Diabetes Prevalence Estimates for 2019 and Projections for 2030 and 2045: Results from the International Diabetes Federation Diabetes Atlas, 9th Edition, 9(th) edition. Diabetes Res. Clin. Pract., 2019. 157:107843. 10.1016/j.diabres.2019.107843 31518657

[B21] Shepherd P. R. Gnudi L. Tozzo E. Yang H. Leach F. Kahn B. B. (1993). Adipose Cell Hyperplasia and Enhanced Glucose Disposal in Transgenic Mice Overexpressing GLUT4 Selectively in Adipose Tissue. J. Biol. Chem. 268, 22243–22246. 10.1016/s0021-9258(18)41516-5 8226728

[B22] Shirakabe A. Ikeda Y. Sciarretta S. Zablocki D. K. Sadoshima J. (2016). Aging and Autophagy in the Heart. Circ. Res. 118, 1563–1576. 10.1161/CIRCRESAHA.116.307474 27174950 PMC4869999

[B23] Sokola-Wysoczanska E. Wysoczański T. Wagner J. Czyż Bodkowski R. Lochyński S. (2018). Polyunsaturated Fatty Acids and Their Potential Therapeutic Role in Cardiovascular System Disorders-A Review. Nutrients 10:1561. 10.3390/nu10101561 PMC6213446 30347877

[B24] Syed I. Lee J. Peroni O. D. Yore M. M. Moraes-Vieira P. M. (2018). Methodological Issues in Studying PAHSA Biology: Masking PAHSA Effects. Cell Metab. 28, 543–546. 10.1016/j.cmet.2018.09.007 30244974 PMC6542592

[B25] Syed I. Lee J. Moraes-Vieira P. M. Donaldson C. J. Sontheimer A. Aryal P. (2018). Palmitic Acid Hydroxystearic Acids Activate GPR40, Which Is Involved in Their Beneficial Effects on Glucose Homeostasis. Cel Metab 27, 419–e4. 10.1016/j.cmet.2018.01.001 PMC5807007 29414687

[B26] Tarquini R. Lazzeri C. Pala L. Rotella C. M. Gensini G. F. (2011). The Diabetic Cardiomyopathy. Acta Diabetol. 48, 173–181. 10.1007/s00592-010-0180-x 20198391

[B27] Voulgari C. Papadogiannis D. Tentolouris N. (2010). Diabetic Cardiomyopathy: from the Pathophysiology of the Cardiac Myocytes to Current Diagnosis and Management Strategies. Vasc. Health Risk Manag. 6, 883–903. 10.2147/VHRM.S11681 21057575 PMC2964943

[B28] Xu L. Brink M. (2016). mTOR, Cardiomyocytes and Inflammation in Cardiac Hypertrophy. Biochim. Biophys. Acta 1863, 1894–1903. 10.1016/j.bbamcr.2016.01.003 26775585

[B29] Xu X. Kobayashi S. Chen K. Timm D. Volden P. Huang Y. (2013). Diminished Autophagy Limits Cardiac Injury in Mouse Models of Type 1 Diabetes. J. Biol. Chem. 288, 18077–18092. 10.1074/jbc.M113.474650 23658055 PMC3689952

[B30] Yore M. M. Syed I. Moraes-Vieira P. M. Zhang T. Herman M. A. Homan E. A. (2014). Discovery of a Class of Endogenous Mammalian Lipids with Anti-diabetic and Anti-inflammatory Effects. Cell 159, 318–332. 10.1016/j.cell.2014.09.035 25303528 PMC4260972

[B31] Zeng X. Yu X. Xiao S. Yao H. Zhu J. (2017). Effects of 1,25-dihydroxyvitamin D3 on Pathological Changes in Rats with Diabetic Cardiomyopathy. Lipids Health Dis. 16, 109. 10.1186/s12944-017-0498-2 28595623 PMC5465473

[B32] Zhang Y. Ling Y. Yang L. Cheng Y. Yang P. Song X. (2017). Liraglutide Relieves Myocardial Damage by Promoting Autophagy via AMPK-mTOR Signaling Pathway in Zucker Diabetic Fatty Rat. Mol. Cel Endocrinol 448, 98–107. 10.1016/j.mce.2017.03.029 28363742

