# Corrigendum: 9-PAHSA Improves Cardiovascular Complications by Promoting Autophagic Flux and Reducing Myocardial Hypertrophy in Db/Db Mice

**DOI:** 10.3389/fphar.2021.827490

**Published:** 2022-01-03

**Authors:** Yan-Mei Wang, Shou-Ling Mi, Hong Jin, Qi-Lin Guo, Zhong-Yu Yu, Jian-Tao Wang, Xiao-Ming Zhang, Qian Zhang, Na-Na Wang, Yan-Yan Huang, Hou-Guang Zhou, Jing-Chun Guo

**Affiliations:** ^1^ Department of Geriatrics of Huashan Hospital, National Clinical Research Center for Aging and Medicine, Department of Translational Neuroscience, Jing’an District Centre Hospital of Shanghai, State Key Laboratory of Medical Neurobiology and MOE Frontiers Center for Brain Science, Institutes of Brain Science, Fudan University, Shanghai, China; ^2^ Department of Cardiology, Zhongshan Hospital, Fudan University, Shanghai, China; ^3^ Shanghai Stomatological Hospital and Institutes of Biomedical Sciences, Fudan University, Shanghai, China

**Keywords:** diabetic cardiovascular complications, autophagy, 9-PAHSA, myocardial hypertrophy, vascular calcification

In the published article, there was an error in affiliation **1** and **4**. Instead of “^1^Department of Geriatrics of Huashan Hospital, National Clinical Research Center for Aging and Medicine, Fudan University, Shanghai, China” and “^4^Department of Translational Neuroscience, Jing’an District Centre Hospital of Shanghai and State Key Laboratory of Medical Neurobiology and MOE Frontiers Center for Brain Science, Institutes of Brain Science, Fudan University, Shanghai, China,” it should be ^*1*^ *Department of Geriatrics of Huashan Hospital, National Clinical Research Center for Aging and Medicine, Department of Translational Neuroscience, Jing’an District Centre Hospital of Shanghai, State Key Laboratory of Medical Neurobiology and MOE Frontiers Center for Brain Science, Institutes of Brain Science, Fudan University, Shanghai, China.*”

The authors apologize for this error and state that this does not change the scientific conclusions of the article in any way. The original article has been updated.

