# Supplementary material for: 9-PAHSA Improves Cardiovascular Complications by Promoting Autophagic Flux and Reducing Myocardial Hypertrophy in Db/Db Mice
Source: Front Pharmacol. 2021 Nov 15;12:754387. doi: 10.3389/fphar.2021.754387 (PMC8634679; doi:10.3389/fphar.2021.754387)
Supplement: Supplementary file 1 [file DataSheet1.docx]

The procedure for the synthesis of 9-PAHSA

1. The synthesis of nonadec-1-en-10-ol:

Into the mixture of magnesium turnings (1.3 g, 52 mmol), elemental iodine (20 mg, ) and THF (100 mL) was added 9-bromonon-1-ene (1.09 g, 5 mmol) under N_2_ atmosphere. The mixture was stirred at 50 ^o^C until the solution turned colorless. The mixture was refluxed and more 9-bromonon-1-ene (9.8 g, 45 mmol) was added slowly. After being refluxed for 0.5 h, the mixture was cooled to room temperature, and was then added dropwise via syringe to the solution of decanal (5.1 g, 33 mmol) in THF (10 mL) at 0 ^o^C. The resulting mixture was warmed to room temperature slowly and stirred overnight. The reaction was quenched by the addition of a saturated solution of ammonium chloride (1 mL). The mixture was concentrated and the pure product was isolated by flash column chromatography (5.58 g, 60% yield). ^1^H NMR (400 MHz, CDCl_3_) δ 5.79 (ddt, *J* = 17.0, 10.3, 6.7 Hz, 1H), 5.01 – 4.89 (m, 2H), 3.60-3.52 (m, 1H), 2.06-1.98 (m, 2H), 1.45 – 1.23 (m, 29H), 0.86 (t, *J* = 6.8 Hz, 3H).

1. The synthesis of nonadec-1-en-10-yl palmitate:

To a stirred solution of nonadec-1-en-10-ol (5.7 g, 20 mmol) in CH_2_Cl_2_ (100 mL) were added palmitic anhydride (12 g, 24 mmol), 4-(dimethylamino)pyridine (1.22 g, 10 mmol), and triethylamine (11 mL, 80 mmol). The solution was stirred for 16 hours at room temperature. The reaction was concentrated and the pure product was isolated by flash column chromatography (7.0 g, 67% yield). ^1^H NMR (400 MHz, CDCl_3_) δ 5.84 – 5.72 (m, 1H), 5.00 – 4.87 (m, 2H), 4.86-4.80 (m, 1H), 2.25 (t, *J* = 7.5 Hz, 2H), 2.05-1397 (m, 2H), 1.63 – 1.56 (m, 2H), 1.52-1.43 (m, 4H), 1.38 – 1.18 (m, 48H), 0.85 (t, *J* = 6.7 Hz, 6H).

1. The synthesis of 1-oxooctadecan-9-yl palmitate:

Ozone was bubbled into a stirred solution of nonadec-1-en-10-ol palmitate (2.1 g, 4 mmol) in CH_2_Cl_2_ (100 mL) at -78 °C until the solution turned blue. Nitrogen was then bubbled into the reaction until it was colorless and triphenyl phosphine (2.1 g, 8 mmole) was added and the reaction was warmed to room temperature. After 2 hours, the mixture was concentrated and the pure product was isolated by flash column chromatography (1.6 g, 80% yield). ^1^H NMR (400 MHz, CDCl_3_) δ 9.74 (t, *J* = 1.6 Hz, 1H), 4.87-4.80 (m, 1H), 2.39 (t, *J* = 7.0 Hz, 2H), 2.25 (t, *J* = 7.8 Hz, 2H), 1.64-1.53 (m, 4H), 1.53-1.40 (m, 4H), 1.38-1.04 (m, 46H), 0.85 (t, *J* = 6.3 Hz, 6H).

1. The synthesis of 9-PAHSA:

Into the solution of 1-oxooctadecan-9-yl palmitate (1.5 g，2.9 mmol) in THF (5 mL) was added the solution of NaH_2_PO_4_ (1.5 g, 12.5 mmol) in water (20 mL). The mixture was cooled to 0 ^o^C and NaClO_2_ (3 g, 33.2 mmol) was added. The resulting mixture was warmed to room temperature and stirred at this temperature for 4 h. After the starting material was completely consumed as monitored by TLC, the solvent was removed by concentration. The remaining viscous aqueous solution was diluted with with ethyl acetate (20 mL). Into the mixture was added aqueous HCl (1 M) until the pH value of the aqueous phase was adjusted to 3 ~ 5. The product was extracted with ethyl acetate (20 mL × 3). The combined organic phase was washed sequentially with water, sat. NaCl aqueous solution, and dried with Na_2_SO_4_. After filtration, the solvent was removed by concentration to give the final product (1.38 g, 89%). ^1^H NMR (400 MHz, CDCl_3_) δ 4.88 – 4.79 (m, 1H), 2.31 (t, *J* = 7.5 Hz, 2H), 2.25 (t, *J* = 7.5 Hz, 2H), 1.64-1.54 (m, 4H), 1.52-1.43(m, 4H), 1.33 – 1.19 (m, 46H), 0.85 (t, *J* = 6.7 Hz, 6H).

**^1^H NMR:**
